# Lactic Acid Bacteria May Impact Intestinal Barrier Function by Modulating Goblet Cells

**DOI:** 10.1002/mnfr.201700572

**Published:** 2018-02-26

**Authors:** Chengcheng Ren, Jelleke Dokter‐Fokkens, Susana Figueroa Lozano, Qiuxiang Zhang, Bart J. de Haan, Hao Zhang, Marijke M. Faas, Paul de Vos

**Affiliations:** ^1^ Immunoendocrinology Division of Medical Biology Department of Pathology and Medical Biology University of Groningen and University Medical Center Groningen Hanzeplein 1 The Netherlands; ^2^ School of Food Science and Technology Jiangnan University Wuxi China

**Keywords:** bioactive food components, goblet cells, intestinal mucus barrier, lactic acid bacteria, modulatory property

## Abstract

**Scope:**

Lactic acid bacteria (LAB) are recognized to promote gastrointestinal health by mechanisms that are not fully understood. LABs might modulate the mucus and thereby enhance intestinal barrier function. Herein, we investigate effects of different LAB strains and species on goblet cell genes involved in mucus synthesis.

**Methods and results:**

Gene expression profiles of goblet‐cell‐associated products (mucin MUC2, trefoil factor 3, resistin‐like molecule β, carbohydrate sulfotransferase 5, and galactose‐3‐O‐sulfotransferase 2) induced by LAB or their derived conditioned medium in human goblet cell line LS174T are studied. Effects of LAB on gene transcription are assessed with or without exposure to TNF‐α, IL‐13, or the mucus damaging agent tunicamycin. LAB do impact the related genes in a species‐ and strain‐specific fashion and their effects are different in the presence of the cytokines and tunicamycin. Bioactive factors secreted by some strains are also found to regulate goblet cell‐related genes.

**Conclusion:**

Our findings provide novel insights in differences in modulatory efficacy on mucus genes between LAB species and strains. This study further unravels direct interactions between LAB and intestinal goblet cells, and highlights the importance of rationally selecting appropriate LAB candidates to achieve specific benefits in the gut.

## 1 Introduction

The intestinal mucus layer serves as a defensive barrier against luminal microbiota and harmful antigens. It is overlying the intestinal epithelium and plays a pivotal role in maintaining homeostasis in the intestine.^1^ Mucin, a family of glycosylated proteins, and especially MUC2, is the predominant component of mucus, and is mainly produced by a type of secretory epithelial cells in the intestine named goblet cells.^1^, ^2^ In the small intestine, the mucus is a single loose layer but in the colon it is organized in a more complex fashion.^1^, ^3^ It was recently shown that the colonic mucus layer in rodents is dependent on the luminal content and is different in the distal and proximal colon.^3^ In the distal colon with fecal pellets, a sterile mucus layer surrounds microbiota‐containing fecal pellets, while in the empty distal colon, loose bacteria‐free mucus exists.^3^ Mucus mixed with microbes attaching to epithelial cells were observed in the proximal colon.^3^ The barrier function of the mucus is determined by quantitative production of mucin but also by glycosylation,^1^ sulfation,^4^, ^5^ and secretion of other mucus‐associated components such as antimicrobial molecules, trefoil factor 3 (TFF3), and resistin‐like molecule β (RELMβ).^1^, ^6^ Together these molecules form a highly‐organized fishnet‐like structure that provides a semi‐permeable barrier between the gut epithelium and the hazardous luminal content.

Mucus structure and thickness can be influenced by several factors including toxins, food products, bile salts, microbial products, hormones, and cytokines.^1^, ^2^, ^7^ These compounds can influence either mucins or other compounds synthesized by intestinal goblet cells such as TFF3 and RELMβ,^1^, ^8^ which are of great importance for maintaining mucosal barrier integrity.^9^, ^10^ It was reported that food‐related mycotoxin deoxynivalenol dampens the expression of RELMβ and mucins via activating protein kinase R and the mitogen‐activated protein kinase p38.^8^ TFF3 is a goblet cell‐derived factor playing a critical role in intestinal mucosal reconstitution and repair, as TFF3 deficiency results in defective healing of intestinal mucosa in mice.^11^ RELMβ, another intestinal goblet cell‐specific protein, has been proposed to be a crucial player in preventing intestinal nematode infection^12^ and attenuating colonic inflammation.^13^ Galactose‐3‐O‐sulfotransferase 2 (GAL3ST2) and carbohydrate sulfotransferase 5 (CHST5), which are expressed in goblet cells,^10^ are implicated in intestinal mucin sulfation. Mucin sulfation is known to reinforce the defensive properties of the mucus barrier against intestinal inflammation and helminth infections.^4^, ^5^ GAL3ST2 catalyzes the transfer of sulfate from 3′‐phosphoadenosine 5′‐phosphosulfate to the C‐3 position of galactose residues, while CHST5 is responsible for transferring sulfate to the C‐6 position of N‐acetylgalactosamine.^10^, ^14^

Dysregulated expression of all the aforementioned genes is associated with defective goblet cell function, which can lead to intestinal diseases. Aberrant goblet cell function comprising impaired TFF3 production, reduction of intestinal goblet cell numbers, and adverse alterations in mucin synthesis, glycosylation, and sulfation is associated with inflammatory bowel disease (IBD).^1^, ^4^ Cytokines released during infection or intestinal disease such as TNF‐α and IL‐13 have been suggested to affect production of mucins and other bioactive molecules by goblet cells.^10^ TNF‐α, a cytokine engaging in the pathogenesis of IBD, was shown to elicit abnormal mucin production in goblet cell lines.^10^ The T helper 2 type cytokine IL‐13 was involved in mediating goblet cell responses to gastrointestinal parasitic infection.^10^, ^12^ Additionally, dampening N‐glycosylation of mucins by tunicamycin (Tm) induces ER stress in goblet cells, leading to disturbed synthesis of mucins.^15^ ER stress is also closely linked to inflammation in IBD.^16^

Bacteria might modulate goblet cell function. A human gut‐associated anaerobic bacterial strain *Ruminococcus gnavus* E1 was shown to augment the expression of mucins (eg., MUC2) in mice mono‐colonized with this strain.^17^ This commensal strain stimulated mucin glycosylation as illustrated by its potentiating effects on the gene expression of glycosyltransferases both in vivo and in vitro.^17^ Recently, we have shown that mucus function and thickness can be modulated by exogenous administration of bacteria.^18^ These bacteria are classified as candidate probiotics as they might contribute to maintenance of intestinal barrier function. The modulating effect of a number of bacterial species was studied in fast ageing *Ercc1^−^* ^/^ *^Δ7^* mice in which decline of the mucus layer is a hallmark of aging. We tested a 10 weeks’ bacterial intervention with *Lactobacillus* (*L*.) *plantarum*, *L. casei*, or *Bifidobacterium* (*B*.) *breve* and assessed effects on gut barrier and mucus thickness. We found that supplementation with *L. plantarum* could prevent age‐associated decline of the mucus layer but that *B. breve* accelerated the decline while *L. casei* was ineffective.^18^ This study illustrates that bioactive food components are able to modulate goblet cell function but that efficacy of putative probiotics is highly species dependent and in some cases even negatively impacts gut homeostasis.

To gain more insight in the species and possible strain‐dependent modulatory properties of lactic acid bacteria (LAB) on goblet cell function, we examined gene expression alterations of some goblet cell‐associated genes (MUC2, TFF3, RETNLB, CHST5, and GAL3ST2) elicited by LAB in the human goblet cell line LS174T. Different LAB strains from various species, which might exert potential beneficial effects on gastrointestinal mucosal barrier functions^18^, ^19^, ^20^, ^21^, ^22^, ^23^, ^24^, ^25^, ^26^ were included in this study to assess their individual effects on expression of genes essential for mucus production in goblet cells. In order to further explore the modulatory potentials of LAB on goblet cell functions under challenged physiological conditions, the effects of LAB on gene expression were also tested when goblet cells were exposed to cytokines (TNF‐α or IL‐13) as well as to the mucus damaging agent Tm. In addition, gene expression profiles induced by stimulation with various LAB strains were compared to gain insight in differences in their regulatory efficacy.

## 2 Experimental Section

### 2.1 Preparation of Bacteria

All bacterial strains used in this study (**Table** 1) were provided by Culture Collections of Food Microbiology (CCFM), and aerobically cultured in De Man‐Rogosa‐Sharpe broth (Merck, Darmstadt, Germany) at 37 °C until reaching stationary phase. Bacterial suspension stocks used for experiments were prepared as previously described.^26^

**Table 1:** Bacterial strains used in this study

Bacterial species	Strain designation	Source or reference
*Lactobacillus plantarum*	CCFM^a^ 634	CGMCC^b^ 9740; Chinese Sichuan pickle isolate
*Lactobacillus plantarum*	CCFM595	CGMCC9511; Chinese Sichuan pickle isolate
*Lactobacillus plantarum*	CCFM382	CGMCC9734; Chinese traditional leavened isolate
*Lactobacillus plantarum*	CCFM675	CGMCC9662; human feces isolate
*Lactobacillus plantarum*	CCFM734	not available
*Lactobacillus fermentum*	CCFM787	not available
*Lactobacillus fermentum*	CCFM381	Chinese traditional leavened isolate
*Lactobacillus fermentum*	CCFM620	Chinese traditional fermented green beans isolate
*Lactobacillus casei*	CCFM9	Pickle isolate
*Lactobacillus casei*	CCFM30	Cow milk isolate
*Lactobacillus reuteri*	CCFM14	CICC^c^ 6226; Yoghurt starter strain
*Lactobacillus rhamnosus*	CCFM237	CGMCC7317
*Lactobacillus acidophilus*	CCFM137	human feces isolate
*Streptococcus thermophilus*	CCFM218	Kefir isolate
*Lactobacillus brevis*	CCFM498	Chinese northeast sauerkraut isolate

a CCFM refers to Culture Collections of Food Microbiology, Jiangnan University, Wuxi, China.

b CGMCC refers to China General Microbiological Culture Collection Center, Beijing, China.

c CICC refers to China Center of Industrial Culture Collection, Beijing, China.

### 2.2 Cell Culture and Reagents

The human colorectal cancer cell line LS174T was obtained from American Type Culture Collection and maintained in MEM eagle medium (Lonza, Verviers, Belgium) supplemented with 10% heat‐inactivated fetal bovine serum (Sigma–Aldrich, St. Louis, MO), 2 mm l‐glutamine (Lonza, Verviers, Belgium), 60 μg mL^–1^ gentamicin sulfate (Lonza, Verviers, Belgium). Cells were cultured at 37 °C in 5% CO_2_ as recommended by the manufacturer. Recombinant human TNF‐α and IL‐13 were obtained from PeproTech (Rocky Hill, NJ). Tm was supplied by Sigma–Aldrich (St. Louis, MO).

### 2.3 Cells Treatment

LS174T cells were resuspended in fresh culture medium at 3 × 10^5^ cells mL^–1^, after which 1 mL of cell suspension was seeded per well in 24‐well plates (Corning, NY). Cells were then cultured until reaching 70–80% confluence. Culture medium was replaced by 1 mL of fresh medium containing different stimuli. For bacterial strains, initial bacterial suspension stocks were diluted with cell culture medium to a final concentration of 2 × 10^7^ CFU mL^–1^ for experiments. LS174T cells were stimulated with either living bacteria, heat‐killed bacteria, or bacteria‐conditioned medium (CM). Heat‐killed bacteria were prepared by heating bacteria at 95 °C for 30 min. Absence of living bacteria was confirmed by plating heat‐killed bacterial samples on De Man‐Rogosa‐Sharpe agar.^27^ Bacterial CM was prepared by culturing bacteria (2 × 10^7^ CFU mL^–1^) in complete cell culture medium for either 12, 24, or 48 h after which bacteria were removed by centrifugation and filtration through 0.2 μm filter (Corning, NY). LS174T cells were treated with bacterial CM obtained from different bacterial incubation period (12, 24, and 48 h). Cell culture medium containing TNF‐α (10 ng mL^–1^), IL‐13 (5 ng mL^–1^) and Tm (1 μg mL^–1^) were used for cytokines and Tm challenge. Cells were incubated with different (combinations of) stimuli for the time periods as indicated in figure captions.

### 2.4 RNA Extraction and Quantitative qRT‐PCR

At the end of stimulation, LS174T cells were homogenized with TRIzol reagent (Life Technologies, Carlsbad). Total RNA was isolated following the manufacturer's instructions, and was reverse transcribed using SuperScript II Reverse Transcriptase (Invitrogen, Carlsbad). qPCR was performed with primer and probe sets (TaqMan Gene Expression Assays) for different genes (MUC2, TFF3, RETNLB, CHST5, GAL3ST2, and GUSB) provided by Applied Biosystems (Foster City, USA) as previously described^9^ and qPCR Mastermix Plus (Eurogentec, Seraing, Belgium). Reactions were carried out in 384‐well PCR plates (Thermo Scientific, UK) using ViiA7 Real‐Time PCR System (Applied Biosystems), and threshold cycle values were calculated by ViiA7 software. Expression levels of target genes were normalized to housekeeping gene GUSB, and comparative quantification of gene expression was analyzed using the 2^−△△Ct^ method.^9^

### 2.5 Statistical Analysis

Data were tested for normality by the Shapiro–Wilk normality test. Statistical comparisons were performed using one‐way analysis of variance with Bonferroni multiple comparisons test for post‐hoc comparison. For stimulation time‐dependent gene expression data, one‐way analysis of variance with Bonferroni multiple comparisons test was conducted for single specific LAB strain to determine significant differences of specific LAB strain at individual time points. GraphPad Prism version 6.0 (San Diego, CA) was used to perform statistical tests. Values of *p* < 0.05 were considered to be statistically significant. Data are presented as mean ± SD. ^#,^*p < 0.05; ^##,^**p < 0.01; ^###,^***p < 0.001.

## 3 Results

### 3.1 LAB Induced Time‐Dependent Modulation of Goblet Cell‐Associated Genes Expression

To investigate whether LAB can modulate goblet cell function and whether their effects are dependent on stimulation time periods, mRNA expression levels of mucus synthesis related genes (MUC2, TFF3, RETNLB, CHST5, and GAL3ST2) in LAB‐treated LS174T cells were analyzed.

**Figure 1:**
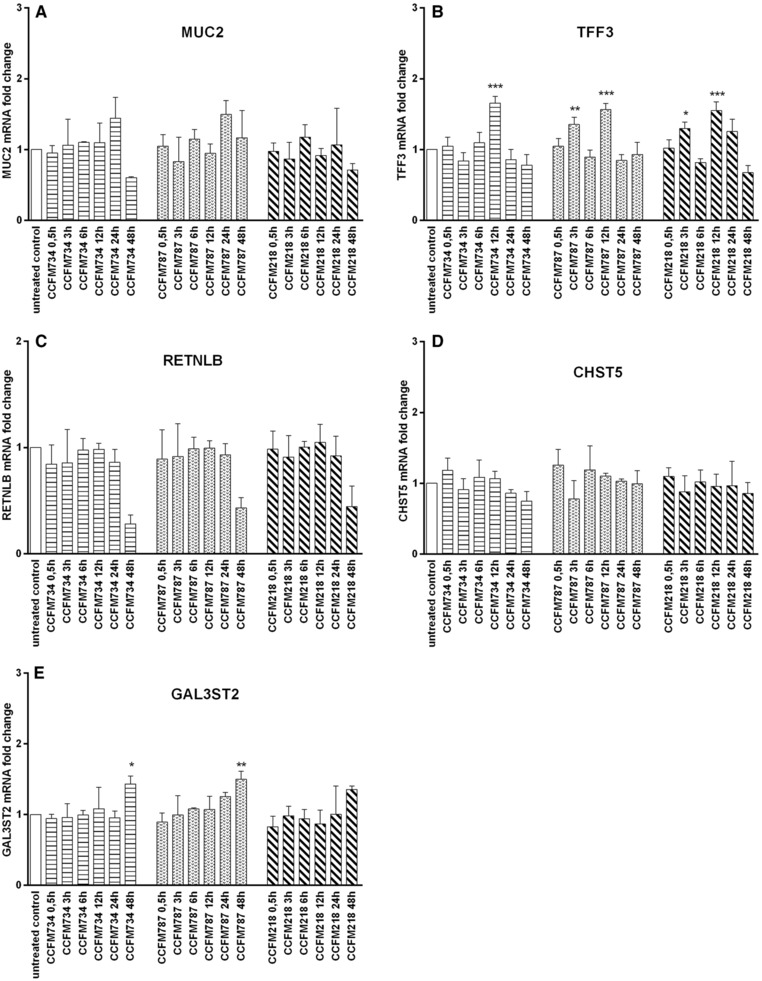
Time‐dependent modulation of goblet cell‐related gene expression in LS174T cells induced by LAB. LS174T cells were treated with different LAB strains (2 × 10^7^ CFU mL^–1^). Expression of MUC2, TFF3, RETNLB, CHST5, and GAL3ST2 was quantified by assessing the mRNA expression with real‐time RT‐PCR at 0.5, 3, 6, 12, 24, and 48 h. Results are presented as fold change against untreated control cells under the same stimulation time period. The results shown represent mean and SD of three independent experiments. Statistical significance between different treatment groups and untreated control group was measured using one‐way analysis of variance with Bonferroni multiple comparisons test (*p < 0.05; **p < 0.01, ***p < 0.001).

Toll‐like receptor (TLR) 2 signaling has been proposed to play a vital role in maintaining mucosal homeostasis.^28^ Therefore, to determine the time‐dependent kinetics of goblet cell modulation, three out of the 15 LAB strains from different species (*L. plantarum*, *L. fermentum*, and *Streptococcus (S.) thermophilus*) which were shown to activate TLR‐signaling pathway and possibly modulate barrier function^26^ were first selected to treat LS174T cells. MUC2, TFF3, RETNLB, CHST5, and GAL3ST2 expression was then studied after LAB stimulation for 0.5, 3, 6, 12, 24, and 48 h. Overall, as shown in **Figure** 1, different LAB strains possessed differential modulatory potentials on transcription of goblet cell‐related genes, and their effects are time‐dependent. TFF3 gene expression was significantly induced after, as early as 3 h of treatment in case of *L. fermentum* CCFM787 *and S. thermophilus* CCFM218 (*p* < 0.05, *p* < 0.01), and it peaked following 12 h of LAB stimulation (*p* < 0.001; Figure 1B). It was observed that mRNA expression of GAL3ST2 generally showed an increased trend in response to LAB between 0.5 and 48 h, and the most significant transcription level was achieved at 48 h of posttreatment with *L. plantarum* CCFM734 *and L. fermentum* CCFM787 (*p* < 0.05, *p* < 0.01; Figure 1E). Moreover, it seemed that for MUC2 a trend toward slightly increased gene expression appeared at 24 h stimulation with *L. plantarum* CCFM734 *and L. fermentum* CCFM787 (Figure 1A). From the above results, 12, 24, and 48 h were selected for further comparative studies of LABs.

### 3.2 Goblet Cell‐Associated Gene Expression was Differentially Modulated by LAB in Species‐ and Strain‐Dependent Manners

**Figure 2:**
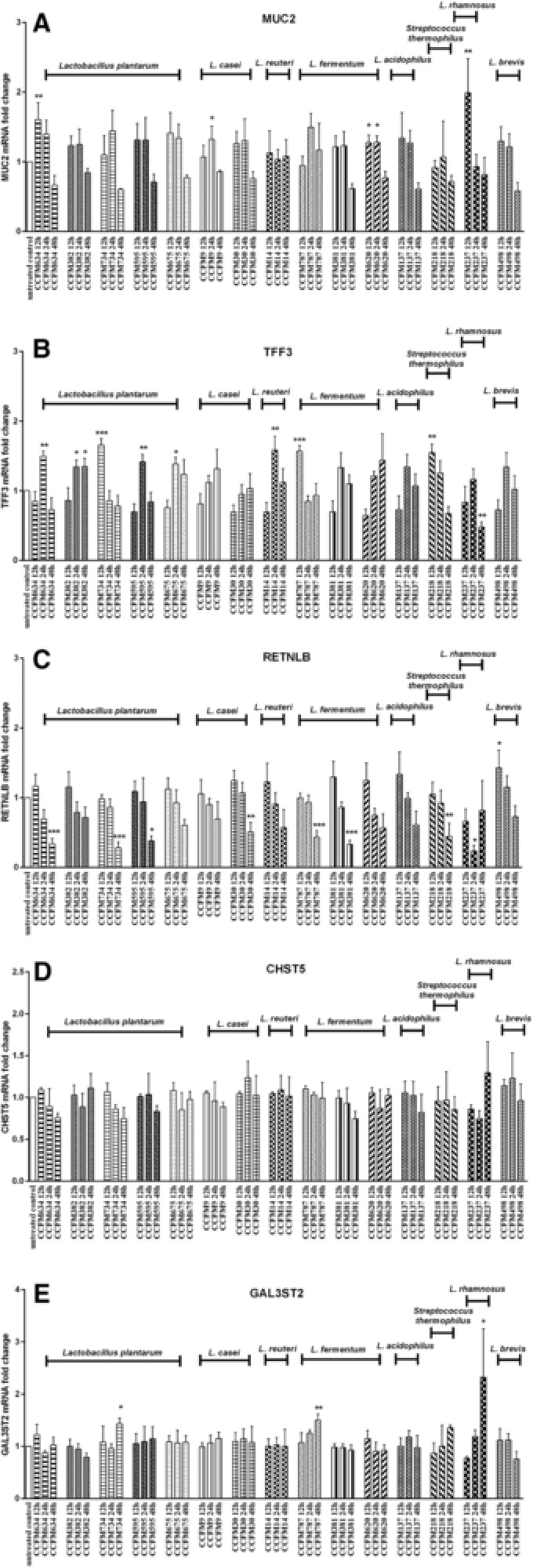
LAB elicited species‐ and strain‐dependent differential gene expression in LS174T cells. LS174T cells were stimulated with various LAB strains (2 × 10^7^ CFU mL^–1^) for 12, 24, and 48 h, after which MUC2, TFF3, RETNLB, CHST5, and GAL3ST2 gene expression was measured by real‐time RT‐PCR. Results are presented as fold change against untreated control cells under the same stimulation time period. The results shown represent mean and SD of three independent experiments. Statistical significance between different treatment groups and untreated control group was measured using one‐way analysis of variance with Bonferroni multiple comparisons test (*p < 0.05; **p < 0.01, ***p < 0.001).

In order to further explore the regulatory effects of LAB strains on mucus synthesis‐related gene expression, 15 LAB strains from various species with potential beneficial effects on gastrointestinal mucosal barrier function^18^, ^19^, ^20^, ^21^, ^22^, ^23^, ^24^, ^25^, ^26^ were examined for their individual impact. We found that different LAB species elicited differential gene expression patterns. For instance, only the species *L. brevis* could strikingly upregulate RETNLB transcription following 12 h of treatment (*p* < 0.05), while LAB strains of other species did not induce a significant increase of expression for this gene (**Figure** 2C). Moreover, it seemed that after 12 h of LAB stimulation a trend toward declining RETNLB expression was elicited by most bacterial species, and significantly decreased RETNLB expression was observed at 48 h of post‐stimulation with the species *L. plantarum, L. casei*, and *L. fermentum* (*p* < 0.05, *p* < 0.01, *p* < 0.001; Figure 2C). The species *L. casei* only significantly augmented MUC2 expression (*p* < 0.05; Figure 2A), whereas no remarkable inductions of expression for other genes were found in response to this species (Figure 2B–E). Similarly, we observed that the species *L. reuteri* only significantly elevated TFF3 expression among all the genes tested (*p* < 0.01; Figure 2B). Besides, there were no pronounced alterations of gene expression levels with treatment by the *L. acidophilus* species (Figure 2).

LAB strains within the same species differentially regulated the intensity of gene expression. For example, the majority of *L. fermentum* strains, such as *L. fermentum* CCFM381 and *L. fermentum* CCFM620 could not trigger significantly increased levels of TFF3 expression, whereas a remarkable increase of TFF3 transcription was observed with stimulation by *L. fermentum* CCFM787 (*p* < 0.001) (Figure 2B). Moreover, the impact of LAB treatment duration on gene expression modification differed among LAB strains within the same species. For example, moderately enhanced TFF3 expression was achieved with an increase in stimulation time for *L. fermentum* CCFM620 whereas the opposite effect was achieved with *L. fermentum* CCFM787 at longer stimulation times. Additionally, TFF3 expression level seemed to peak at 24 h of posttreatment with *L. fermentum* CCFM381 and thereafter it declined (Figure 2B). A similar strain‐dependent and time kinetics of gene expression alteration was also observed in the species *L. plantarum*. Of note, specific LAB strains seemed to influence specific genes. For instance, *L. rhamnosus* CCFM237 specifically elevated MUC2 and GAL3ST2 expression (*p* < 0.05, *p* < 0.01; Figure 2A and E) and not the other genes. Also for some other LAB strains such as *L. plantarum* CCFM634, expression of MUC2 and TFF3 was specifically enhanced with differential time kinetics (Figure 2A and B).

### 3.3 LAB‐CM But not Heat‐Killed LAB Distinctively Regulated Goblet Cell‐Associated Gene Expression in Species‐ and Strain‐Dependent Patterns

**Figure 3:**
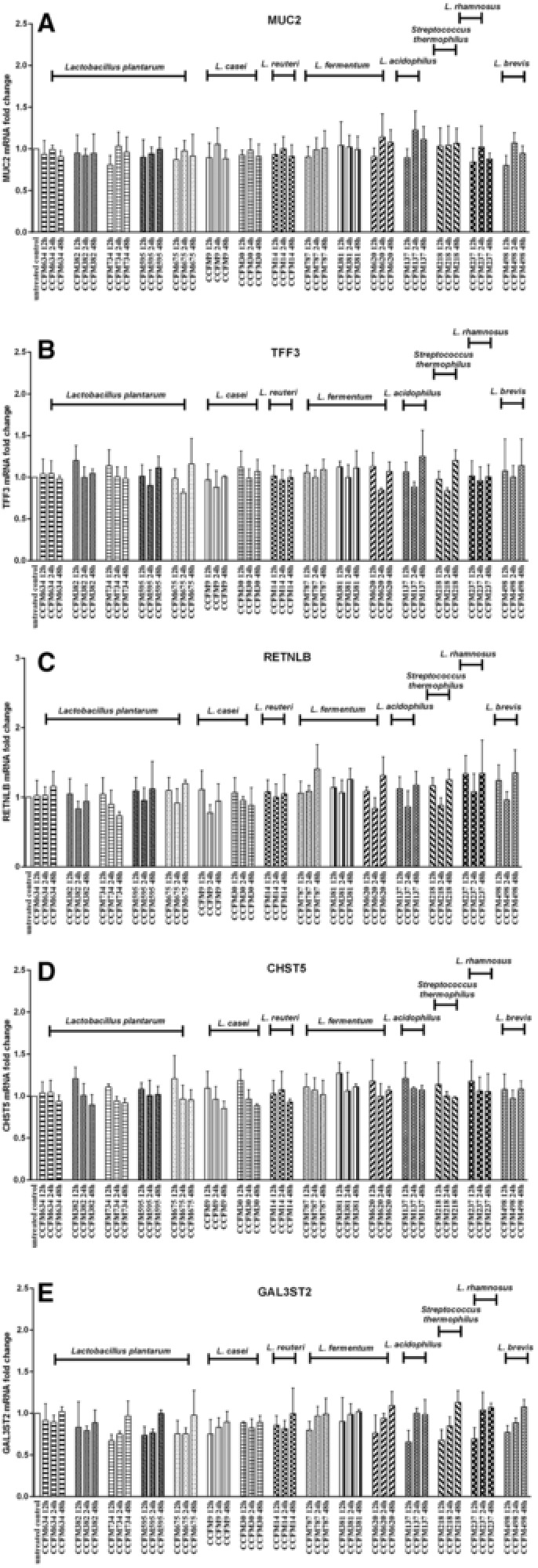
Heat‐killed LAB did not regulate mucus function‐associated gene expression in LS174T cells. LS174T cells were stimulated with heat‐killed LAB strains (2 × 10^7^ CFU mL^–1^) for 12, 24, and 48 h, after which MUC2, TFF3, RETNLB, CHST5, and GAL3ST2 gene expression was measured by real‐time RT‐PCR. Results are presented as fold change against untreated control cells under the same stimulation time period. The results shown represent mean and SD of three independent experiments. Statistical significance between different treatment groups and untreated control group was measured using one‐way analysis of variance with Bonferroni multiple comparisons test (*p < 0.05; **p < 0.01, ***p < 0.001).

To determine whether LABs need to be alive to exert modulatory capacities on goblet cell function, goblet cell‐associated gene expression profiles induced by heat‐killed LAB were measured in a separate set of experiments. However, we observed no significant gene expression change induced by heat‐killed LAB (**Figure** 3A–E).

**Figure 4:**
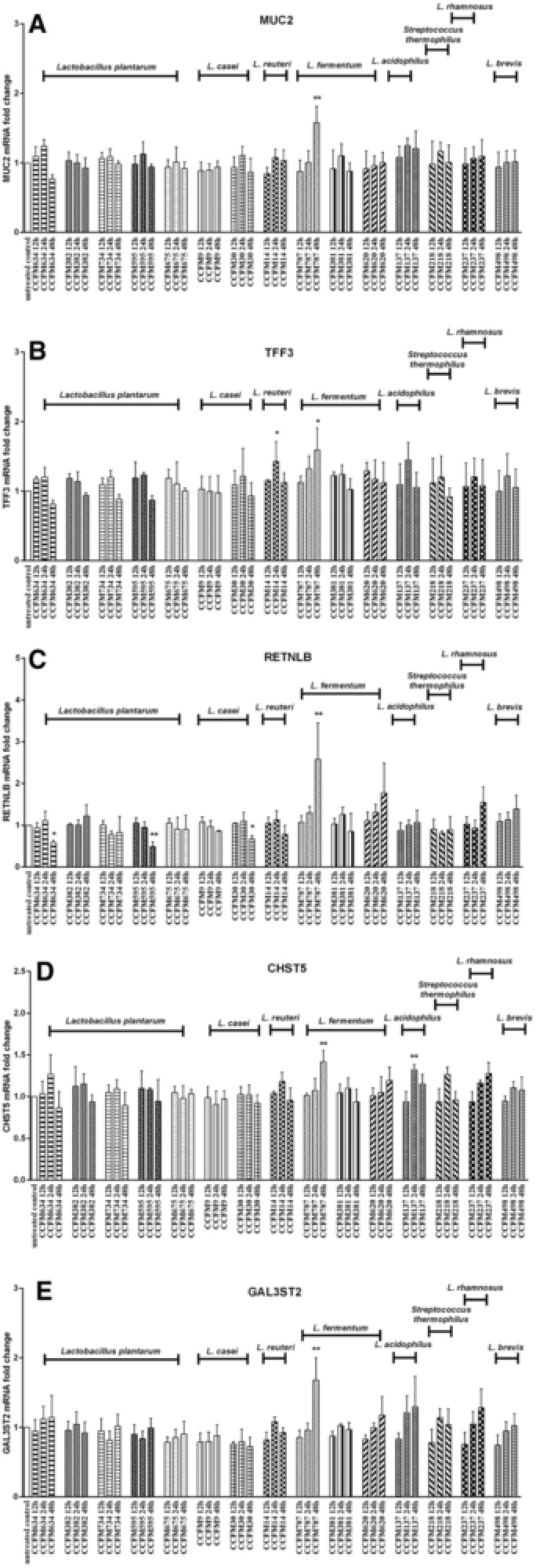
LAB‐derived conditioned medium (CM) elicited species‐ and strain‐dependent gene expression in LS174T cells. LS174T cells were incubated with various LAB‐CM collected from different bacterial culture period (12, 24, and 48 h) for corresponding time period (12, 24, and 48 h), after which MUC2, TFF3, RETNLB, CHST5, and GAL3ST2 gene expression was measured by real‐time RT‐PCR. Results are presented as fold change against untreated control cells under the same stimulation time period. The results shown represent mean and SD of three independent experiments. Statistical significance between different treatment groups and untreated control group was measured using one‐way analysis of variance with Bonferroni multiple comparisons test (*p < 0.05; **p < 0.01, ***p < 0.001).

Next, we determined whether it was solely LAB‐goblet cell interactions that were responsible for the observed effects on goblet cells or whether also factors released by the bacteria in medium can have such effects. To this end, CM was collected of bacteria cultured for 12, 24, and 48 h and exposed to LS174T cells. Overall, as shown in **Figure** 4, regulatory effects of LAB‐CM were observed. We found that treatment with LAB‐CM exerted species‐ and strain‐specific effects on goblet cell‐related genes, similar to that were observed with direct LAB‐goblet cell interactions. CM from *L. fermentum* CCFM787 upregulated MUC2 and GAL3ST2 expression following 48 h of incubation, while no significant expression change of these two genes was observed with CM from other *L. fermentum* strains or strains of other species (*p* < 0.01; Figure 4A and E). In addition, CM from *L. fermentum* CCFM787 also elicited significant enhancement in transcription of TFF3, RETNLB, and CHST5 (*p* < 0.05, *p* < 0.01; Figure 4B–D). Notably, CM from *L. reuteri* CCFM14 was found to significantly augment TFF3 transcription at 24 h (*p* < 0.05; Figure 4B), which was similar to what was observed with the direct interaction of this strain with goblet cells (*p* < 0.01; Figure 2B). Similarly, for several strains such as *L. plantarum* CCFM634, *L. plantarum* CCFM595, and *L. casei* CCFM30 RETNLB expression was significantly attenuated by both these strains and their CM (*p* < 0.05, *p* < 0.01, *p* < 0.001; Figures 2C and 4C).

### 3.4 LAB Strains Differentially Modulated Transcription of Mucus Synthesis Genes During TNF‐α or IL‐13 Challenge

**Figure 5:**
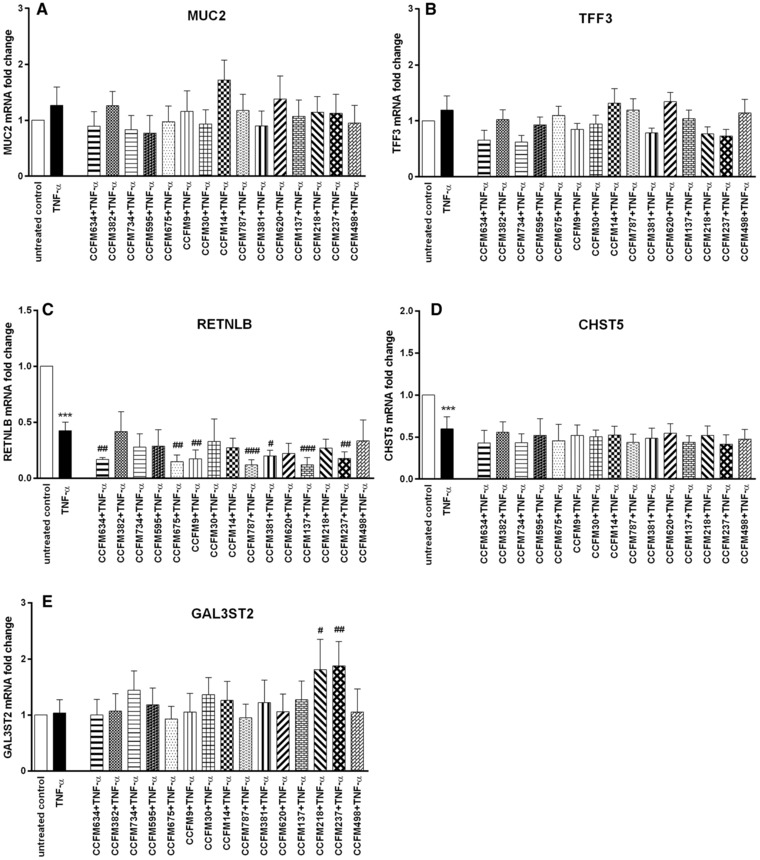
LAB elicited differential gene expression change in LS174T cells during TNF‐α challenge. MUC2, TFF3, RETNLB, CHST5, and GAL3ST2 gene expression in LS174T cells was measured by real‐time RT‐PCR following simultaneous stimulation with various LAB strains (2 × 10^7^ CFU mL^–1^) together with TNF‐α (10 ng mL^–1^) for 48 h. Results are presented as fold change against untreated control cells. The results shown represent mean and SD of five independent experiments. Statistical significance was measured using one‐way analysis of variance with Bonferroni multiple comparisons test (* vs untreated control; ^#^ vs TNF‐α group; ^#,^*p < 0.05; ^##,^**p < 0.01; ^###,^***p < 0.001).

Next, we investigated the effects of different LAB strains on goblet cell‐associated genes when simultaneously exposed to the cytokines TNF‐α or IL‐13. These cytokines are known to influence goblet cell function.^10^ As shown in **Figure** 5, TNF‐α significantly inhibited RETNLB and CHST5 expression (*p* < 0.001 vs untreated control). LAB did not effectively upregulate the expression of RETNLB and CHST5 in the presence of TNF‐α, while several LAB strains such as *L. plantarum* CCFM634, *L. fermentum* CCFM787, and *L. acidophilus* CCFM137 were found to induce a further decreased RETNLB expression in the presence of TNF‐α (*p* < 0.01, *p* < 0.001 vs TNF‐α; Figure 5C). Furthermore, for the two genes MUC2 and TFF3, whose transcription was not significantly affected by TNF‐α stimulation, LAB also could not enhance the expression of these two genes (Figure 5A and B). However, LAB‐specific rescuing effects were observed for *S. thermophilus* CCFM218 and *L. rhamnosus* CCFM237 during TNF‐α stimulation which strikingly enhanced GAL3ST2 expression (*p* < 0.05, *p* < 0.01 vs TNF‐α; Figure 5E).

**Figure 6:**
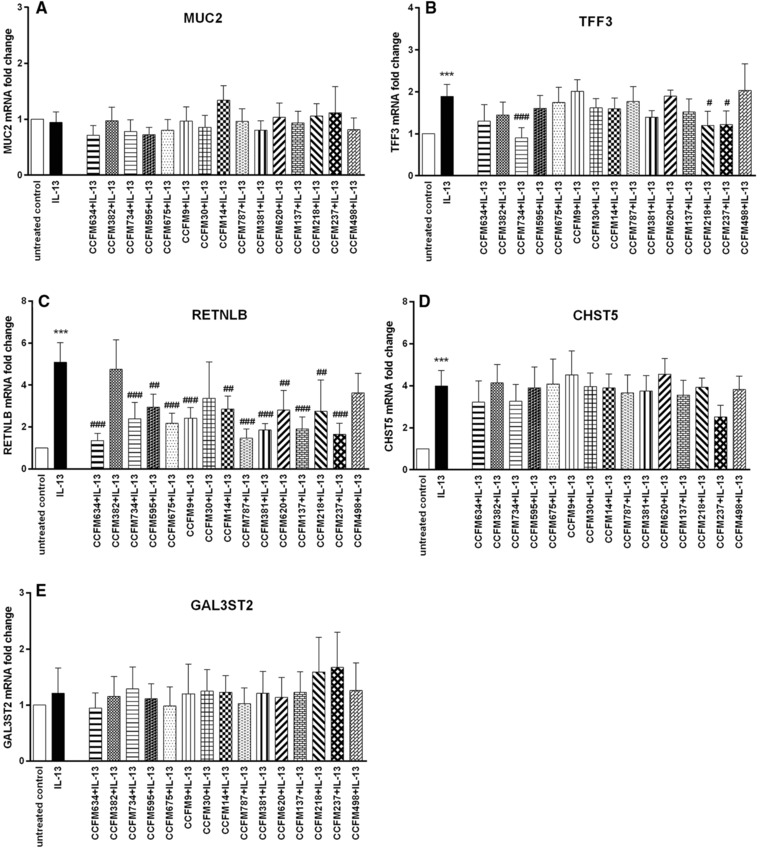
LAB elicited differential gene expression change in LS174T cells during IL‐13 challenge. MUC2, TFF3, RETNLB, CHST5, and GAL3ST2 gene expression in LS174T cells was measured by real‐time RT‐PCR following concomitant treatment with various LAB strains (2 × 10^7^ CFU mL^–1^) together with IL‐13 (5 ng mL^–1^) for 48 h. Results are presented as fold change against untreated control cells. The results shown represent mean and SD of five independent experiments. Statistical significance was measured using one‐way analysis of variance with Bonferroni multiple comparisons test (*vs untreated control; ^#^vs IL‐13 group; ^#,^*p < 0.05; ^##,^**p < 0.01; ^###,^***p < 0.001).

IL‐13 has different effects on goblet cells than TNF‐α. IL‐13 treatment potentiated the expression of TFF3, RETNLB, and CHST5 (*p* < 0.001 vs untreated control). During IL‐13 stimulation the expression of TFF3, RETNLB, and CHST5 was not upregulated by LAB (**Figure** 6B–D). However, some LAB strains such as *L. plantarum* CCFM734, *S. thermophilus* CCFM218, and *L. rhamnosus* CCFM237 even lowered TFF3 expression during IL‐13 exposure (*p* < 0.05, *p* < 0.001 vs IL‐13), and these strains seemed to regulate IL‐13‐elicited elevated TFF3 expression to the level closer to normal expression level (Figure 6B). In addition, most LAB strains except *L. plantarum* CCFM382, *L. casei* CCFM30, and *L. brevis* CCFM498 were also observed to profoundly inhibit IL13‐triggered heightened RETNLB expression (*p* < 0.01, *p* < 0.001 vs IL‐13; Figure 6C). Moreover, IL‐13 exposure did not influence MUC2 and GAL3ST2 transcription, and unaltered expression of these two genes was found with LAB treatment under IL‐13 stimulation (Figure 6A and E).

Overall, different LAB strains could differentially modulate different mucus synthesis genes during TNF‐α or IL‐13 challenge.

### 3.5 LAB Did not Restore Tm‐Induced Declined Gene Expression of Mucus Synthesis Genes

**Figure 7:**
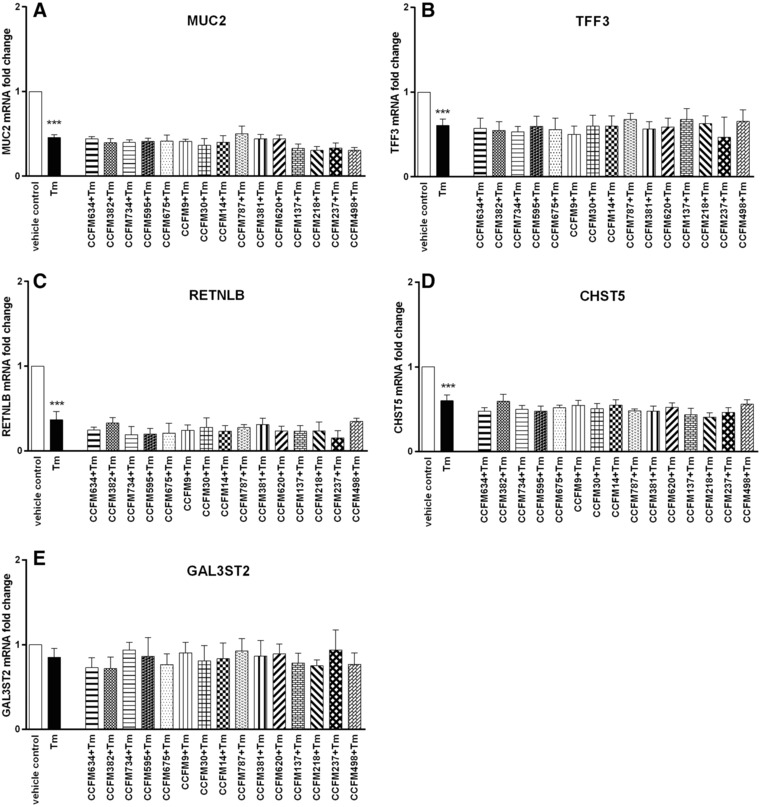
LAB did not abrogate Tm‐induced impaired gene expression of goblet cell‐associated products in LS174T cells. LS174T cells were first pretreated with various LAB strains (2 × 10^7^ CFU mL^–1^) for 24 h, after which cells were exposed to Tm for another 24 h. MUC2, TFF3, RETNLB, CHST5, and GAL3ST2 gene expression in LS174T cells was measured by real‐time RT‐PCR following Tm stimulation. Results are presented as fold change against vehicle‐treated control cells. The results shown represent mean and SD of five independent experiments. Statistical significance was measured using one‐way analysis of variance with Bonferroni multiple comparisons test (*vs vehicle control; ^#^vs Tm group; ^#,^*p < 0.05; ^##,^**p < 0.01; ^###,^***p < 0.001).

Tm is a N‐glycosylation inhibitor and known to disrupt mucus synthesis in goblet cells.^29^ To examine the protective properties of LAB on Tm‐induced disruption of goblet cell function, LS174T cells were stimulated with Tm for 24 h after 24 h of pretreatment with various LAB strains. This setup of pre‐exposure to LAB was chosen based on a previous report that preventative treatment exerted the most effective protection against Tm‐induced ER stress in goblet cells.^15^ As shown in **Figure** 7, Tm treatment significantly suppressed the expression of MUC2, TFF3, RETNLB, and CHST5 (*p* < 0.001 vs vehicle control). Pretreatment with various LAB strains could not prevent Tm‐elicited dampened expression of these genes. Moreover, significantly diminished GAL3ST2 expression was not found with Tm challenge, and its expression also could not be effectively modulated by LAB (Figure 7E). The above results suggest that LAB was not capable to suppress Tm‐elicited impaired expression of mucus synthesis genes in goblet cells.

## 4 Discussion

**Figure 8:**
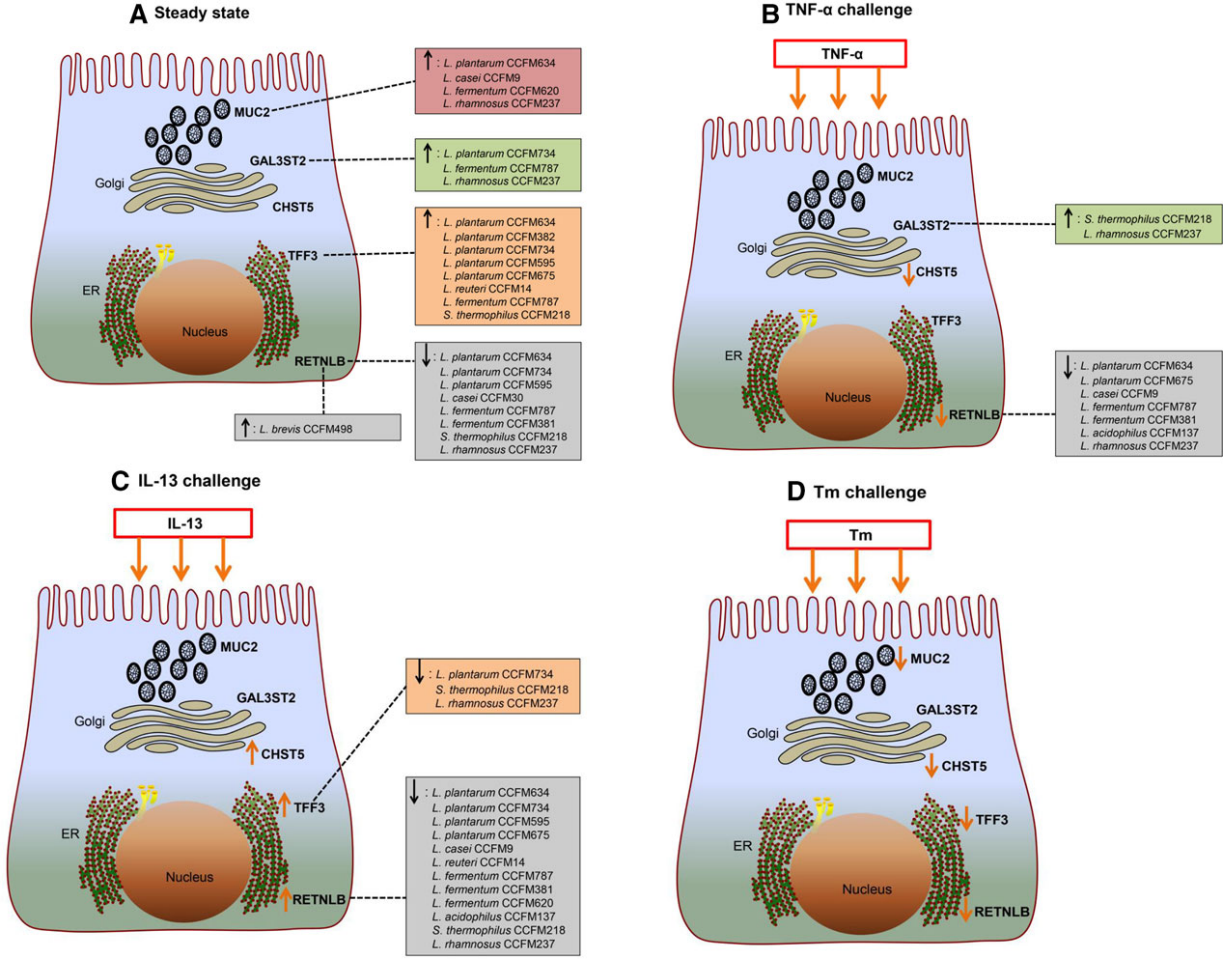
Schematic illustrating modulation of goblet cell‐related gene (MUC2, TFF3, RETNLB, CHST5, and GAL3ST2) expression induced by LAB in LS174T goblet cell line. A) Regulation of gene expression by LAB under steady state. B–D) Regulation of gene expression by LAB during exposure to different stimulating agent (TNF‐α, IL‐13, or Tm).

In the present study, we studied species‐ and strain‐specific effects of LABs on goblet cell‐associated genes known to be involved in mucus production and function such as MUC2, TFF3, RETNLB, CHST5, and GAL3ST2.^10^ To the best of our knowledge, this is the first study comparing the goblet‐cell modulatory abilities of various LABs and demonstrating a species but also strain‐dependent effect on goblet cell function. In the absence of any goblet‐cell stimulating agent, effects of LABs were shown to be highly species and strain specific. The rescuing effects of LABs on goblet cells challenged with inflammatory cytokines or a mucus‐disrupting agent were again species‐ and strain‐dependent but also dependent on disrupting agent. With TNF‐α the most pronounced effects were observed with *S. thermophilus* CCFM218 or *L. rhamnosus* CCFM237, which significantly reinforced GAL3ST2 gene expression during TNF‐α challenge. With IL‐13 we only observed inhibition of IL‐13 elicited upregulated TFF3 and RETNLB expression by some LAB strains such as *L. plantarum* CCFM734, *S. thermophilus* CCFM218, and *L. rhamnosus* CCFM237. But for stressor Tm, LAB strains did not exert rescuing effects on Tm‐induced defective transcription of mucus synthesis genes. The differential effects of various bacterial strains on the different studied mucus pathways in goblet cells are summarized in **Figure** 8.

Effects of LABs on goblet cell function were first assessed under homeostatic conditions, and were demonstrated to be species‐ and strain‐specific. A representative gene regulated by LAB is TFF3, which is a secretory peptide of intestinal goblet cells, and involved in mucosal healing and regeneration.^1^ Its expression was markedly heightened by several LAB strains such as *L. plantarum*, *L. reuteri*, *L. fermentum*, and *S. thermophilus*, indicating that these TFF3‐promoting LAB strains have the potential to facilitate intestinal mucosal restitution and healing and thereby contribute to maintenance of mucus barrier integrity. MUC2, as the fundamental structural constituent of intestinal secreted mucus, is essential for maintaining intestinal health.^30^ *L. plantarum* CCFM634, *L. casei* CCFM9, *L. fermentum* CCFM620, and *L. rhamnosus* CCFM237 enhanced MUC2 expression and therefore are potential candidate strains for strengthening mucus barrier function. RELMβ, another goblet cell‐secreted product with bioactive effects, plays a vital role in fighting against intestinal parasite infection and inflammation.^12^ *L. brevis* CCFM498 was found to specifically potentiate RETNLB transcription, and thus is a candidate conferring beneficial effects against helminth infection and inflammation in the gut. In addition, some LAB strains such as *L. plantarum* CCFM734, *L. fermentum* CCFM787, and *L. rhamnosus* CCFM237 upregulated the transcription of sulfotransferase GAL3ST2. GAL3ST2 is essentially involved in mucin sulfation, which is known to prevent mucin degradation and protects against cancer, inflammation as well as pathogenic infections in the intestine.^4^, ^5^, ^10^ Thus, the above‐mentioned strains are prospective candidates for reinforcing mucus barrier function. Notably, specific LAB strains such as *L. plantarum* CCFM734 increased GAL3ST2 expression but attenuated MUC2 expression at 48 h of post‐stimulation. Although speculative, it is possible that GAL3ST2 expression was potentiated to reinforce the mucus gel with less mucin “bricks” resulting from declined MUC2 expression. Further in‐depth studies are warranted to decipher the regulation network of these different genes within goblet cells. Here stimulation duration‐dependent effects were also observed with LAB. This suggests that in addition to selection of appropriate LAB strains treatment duration is essential in steering mucus reinforcement.

Effects of heat‐killed bacteria and bacterial CM on the expression of goblet cell‐related genes were also investigated. We however did not observe any gene expression changes elicited by heat‐killed LAB, illustrating that living bacteria are needed to elicit effects on goblet cells. This was in line with a previous study which also reports that dead bacteria were not as effective as living bacteria in supporting gut barrier function.^27^ In contrast, CM from some LAB strains significantly regulated the expression of mucus function‐associated genes, illustrating that bacteria might produce specific factors that modulate goblet cell function. For example, CM from *L. fermentum* CCFM787 potentiated transcription of all tested genes following 48 h of stimulation, indicating that bioactive agents secreted by this strain could regulate goblet cell function. Moreover, CM from several LAB strains such as *L. reuteri* CCFM14, *L. plantarum* CCFM634, *L. plantarum* CCFM595, and *L. casei* CCFM30 modified transcription of specific genes (TFF3 and RETNLB) in a similar pattern as the living bacteria, implying that active factors released by these strains are at least partially responsible for the modulation of gene expression. Intriguingly, pronounced regulation of specific genes by certain LAB strains was only achieved with direct living bacteria treatment. For instance, *L. plantarum* strains significantly elevated TFF3 gene expression, which was not observed with either heat‐killed strains or their CM.

Regulatory potentials of LABs were also evaluated in the presence of an inflammatory cytokine challenge. Species‐ and strain‐dependent effects of LAB strains were again observed here. TNF‐α was applied as it is a well‐defined and major inflammatory cytokine in the pathogenesis of IBD, and is linked to compromised goblet cell responses and mucus barrier dysfunction in IBD.^1^, ^10^ During TNF‐α challenge *S. thermophilus* CCFM218 and *L. rhamnosus* CCFM237 significantly potentiated transcription of sulfotransferase GAL3ST2, which makes these two strains promising candidates for promoting mucin sulfation and strengthening mucus barrier. For the strain *L. rhamnosus* CCFM237, its elevating effects on GAL3ST2 expression was also shown in the steady state, which further proves its potential for supporting mucin sulfation. However, it is worth noting that *L. rhamnosus* CCFM237 also induced a declined RETNLB transcription under TNF‐α exposure, indicating that this strain is not a potential suitable candidate for RETNLB targeted restoration of TNF‐α‐induced damage to mucus. On the basis of our data we conclude that *S. thermophilus* CCFM218 is a LAB strain with potential for reinforcing mucus barrier in TNF‐α‐mediated intestinal inflammatory disorder such as IBD. Furthermore, some other LAB strains such as *L. plantarum* CCFM634, *L. plantarum* CCFM675, *L. casei* CCFM9, *L. fermentum* CCFM787, *L. fermentum* CCFM381, and *L. acidophilus* CCFM137 were also found to further reduce RETNLB expression during TNF‐α stimulation, suggesting that it might not be appropriate to apply these strains for RETNLB targeted rescue of TNF‐α‐induced disturbed mucus function.

IL‐13 was used in the current study since it is a key T helper 2 mediator in eliminating intestinal helminth infection via regulating goblet cell function to enhance mucosal barrier function.^1^, ^10^ During IL‐13 challenge, no significant augmentation of goblet cell‐related gene expression was observed with LAB treatment. Moreover, our results demonstrated suppressing effects of some LAB strains on goblet cell‐associated gene expression during IL‐13 stimulation. IL‐13 was found to remarkably augment the expression of genes involved in mucus function (TFF3, RETNLB, and CHST5). Similar as their decreasing effects on RETNLB expression under TNF‐α challenge, *L. plantarum* CCFM634, *L. plantarum* CCFM675, *L. casei* CCFM9, *L. fermentum* CCFM787, *L. fermentum* CCFM381, *L. acidophilus* CCFM137, and *L. rhamnosus* CCFM237 effectively abrogated IL‐13‐induced increase in RETNLB expression, and tended to adjust its expression toward normal expression level. The consistent inhibiting effects of the above mentioned LAB strains on RETNLB expression under both TNF‐α and IL‐13 challenge suggest that both cytokines might exert their impact on RETNLB transcription via the same signaling pathway, and these strains might act on this distinct pathway to influence gene expression. Additionally, similar to the suppressing properties of LAB on IL‐13‐triggered RETNLB expression, attenuation of heightened TFF3 expression elicited by IL‐13 was also achieved with several LAB strains such as *L. plantarum* CCFM634, *S. thermophilus* CCFM218, and *L. rhamnosus* CCFM237.

Tm‐induced mucin glycosylation defects have been suggested to lead to ER stress, which subsequently results in mucus barrier dysfunction and intestinal inflammation.^29^ Thus, in the present study the protective effects of LABs on Tm‐triggered impaired goblet cell function were evaluated to further explore the functional properties of LABs on intestinal mucus barrier. However, we did not observe a modulatory impact of LABs on mucus function‐related gene expression. The above results indicate that LAB strains tested in this study might not be capable for strengthening mucus barrier function via regulating ER stress‐induced abnormal mucus synthesis.

Intriguingly, we found that only one of the two sulfotransferases, GAL3ST2, was specifically modulated by LABs, while the sulfotransferase CHST5 was not influenced by any of the tested LABs. This implies that transcription of these two different sulfotransferases are controlled by different signaling pathways and several LAB strains could only specifically act on GAL3ST2 expression‐associated pathway. Interestingly, we also observed that GAL3ST2 expression was further mounted by *S. thermophilus* CCFM218 and *L. rhamnosus* CCFM237 during exposure to TNF‐α, which was not seen under exposure to other stimulating agents. This suggests that TNF‐α might impact GAL3ST2 expression via different signaling pathways from IL‐13 or Tm, and these two strains might specifically act on TNF‐α‐involved pathway. Furthermore, promoting properties of *L. plantarum* CCFM734 and *L. fermentum* CCFM787 on GAL3ST2 transcription in steady state were absent in the presence of stimulating agents (TNF‐α, IL‐13, or Tm). Taken together, the above observations indicate that modulatory functions of LABs largely depend on specific physiological states.

In conclusion, we demonstrate the potential regulatory properties of LABs on goblet cell function via modulation of mucus barrier function related genes. Despite the fact that mRNA and protein levels are not always in agreement, previous studies showed that transcription and protein levels of MUC2, TFF3, RETNLB, and CHST5 correlated well under similar conditions in LS174T or its derivative cell line.^9^, ^31^ Owing to the specific configuration of intestinal mucus, direct goblet cell–LAB crosstalk most probably occurs in the small intestine and especially in the proximal colon, where mucus–bacteria mixture was observed to be in close contact with epithelium in rodents.^2^, ^3^ This notion also has been confirmed by our previous study in which a *L. plantarum* strain was shown to restore impaired mucus barrier of the proximal colon in a fast aging mouse model.^18^ In this study only one of the tested bacteria was beneficial for mucus restoration.^18^ The current study was undertaken as a follow‐up to identify candidate strains with functional effects on goblet cell activity. We aimed to develop a technology platform as a cost‐ and time‐effective tool for different LAB strains prior to testing in animal models or humans. We selected specific genes known to be involved in mucus function. Just as in our in vivo study^18^ species‐ but also strain‐dependency of LAB‐induced effects on mucus was observed. Moreover, LABs were observed to confer varied modulatory activities on goblet cells in homeostasis or exposed to cytokines or mucus damaging agent. This supports the notion that individual LAB strains exert differential regulatory capacities in healthy and various diseased situations.^32^, ^33^ In this respect, selection and application of suitable LAB candidates for particular clinical purposes is indispensable. Molecular mechanisms involved in the regulatory functions of different LAB strains remain to be further explored in order to better understand the strain specificity of their modulatory activities and to identify appropriate LAB strains for specific target populations.

**Abbreviations**
CHST5: carbohydrate sulfotransferase 5
CM: conditioned medium
GAL3ST2: galactose‐3‐O‐sulfotransferase 2
IBD: inflammatory bowel disease
LAB: lactic acid bacteria
RELMβ: resistin‐like molecule β
TFF3: trefoil factor 3
Tm: tunicamycin

## Conflict of Interest

The authors declare no conflict of interest.
